# Gender context of sexual violence and HIV sexual risk behaviors among married women in Iringa Region, Tanzania

**DOI:** 10.3402/gha.v7.25346

**Published:** 2014-12-05

**Authors:** Tumaini M. Nyamhanga, Gasto Frumence

**Affiliations:** Department of Development Studies, Muhimbili University of Health and Allied Sciences, Dar es Salaam, Tanzania

**Keywords:** Gender, sexual violence, HIV, sexual risk, married women

## Abstract

**Background:**

There is a dearth of empirical research illuminating possible connections between gender imbalances and sexual violence among married women in Tanzania. There is a need to generate in-depth information on the connectivity between gender imbalances (asymmetrical resource ownership, sexual decision making, roles, and norms) and sexual violence plus associated HIV risky sexual behavior among married women.

**Design:**

This paper is based on a qualitative case study that involved use of focus group discussions (FGDs). A thematic analysis approach was used in analyzing the study findings.

**Results:**

The study findings are presented under the three structures of gender and power theory. *On sexual division of labor*, our study found that economic powerlessness exposes women to sexual violence.

*On sexual division of power*, our study found that perception of the man as a more powerful partner in marriage is enhanced by the biased marriage arrangement and alcohol consumption.

*On cathexis*, this study has revealed that because of societal norms and expectations regarding women's sexual behavior characterized by their sexual and emotional attachments to men, women find it hard to leave sexually abusive marriages. That is, because of societal expectations of obedience and compelled tolerance many married women do suffer in silence. They find themselves trapped in marriages that increase their risk of acquiring HIV.

**Conclusions:**

This study suggests that married women experience a sexual risk of acquiring HIV that results from non-consensual sex. That non-consensual sex is a function of gender imbalances – ranging from women's economic dependence on their husbands or partners to socioculturally rooted norms and expectations regarding women's sexual behavior. The HIV risk is especially heightened because masculine sexual norms encourage men [husbands/partners] to engage in unprotected intra- and extramarital sex. It is recommended that the Tanzania Commission for AIDS (TACAIDS) should address the gender dimensions of sexual violence in marriage.

Globally, in the year 2011, there were 2.5 million new HIV infections and 1.7 million AIDS-related deaths (1). Of these, 1.8 million new infections and 1.2 million deaths were recorded in sub-Saharan Africa. Furthermore, about 76% of all HIV-positive women in the world live in this region (2). Unprotected heterosexual intercourse remains the main route of transmission (3). Gender-based violence has been considered an outstanding driver of the epidemic among women in sub-Saharan Africa (4–6). In the recent past, evidence has indicated that intimate partner violence (IPV) is widespread in sub-Saharan African countries (7–9). Durevall and Lindskog (10) analyzed Demographic and Health Survey (DHS) data from eight countries, namely Malawi, Zambia, Zimbabwe, Kenya, Rwanda, Burkinafaso, Mali, and Liberia, and found that 20–50% of married women experienced IPV.

Similarly, research conducted in Tanzania has documented unacceptably high levels of physical and sexual violence against women (11, 12). For instance, a multicountry study sponsored by the World Health Organization reported that 56% of ever-partnered women in Mbeya and 41% in Dar es Salaam had ever experienced physical or sexual violence at the hands of a partner (11). Moreover, McCloskey et al. (12) conducted a study in the urban district of Moshi and reported that 21% of women reported having experienced IPV and that the likelihood of violence in the past year was elevated if the woman's husband engaged in extramarital sexual affairs.

It can be concluded from the literature that the existence of IPV in Tanzania has been well documented (11, 12). However, empirical research illuminating possible connections between gender imbalances on one hand and sexual violence (sex against one's wishes) and associated HIV risky sexual behavior among married women on the other hand remains limited. There is a need to generate in-depth information about the connectivity between gender imbalances (asymmetrical resource ownership, sexual decision making, roles, and norms) and sexual violence plus associated HIV risky sexual behavior among married women. This paper is a contribution toward that direction.

## Theoretical framework: examining women's vulnerability to HIV/AIDS through IPV

**Fig. 1:**
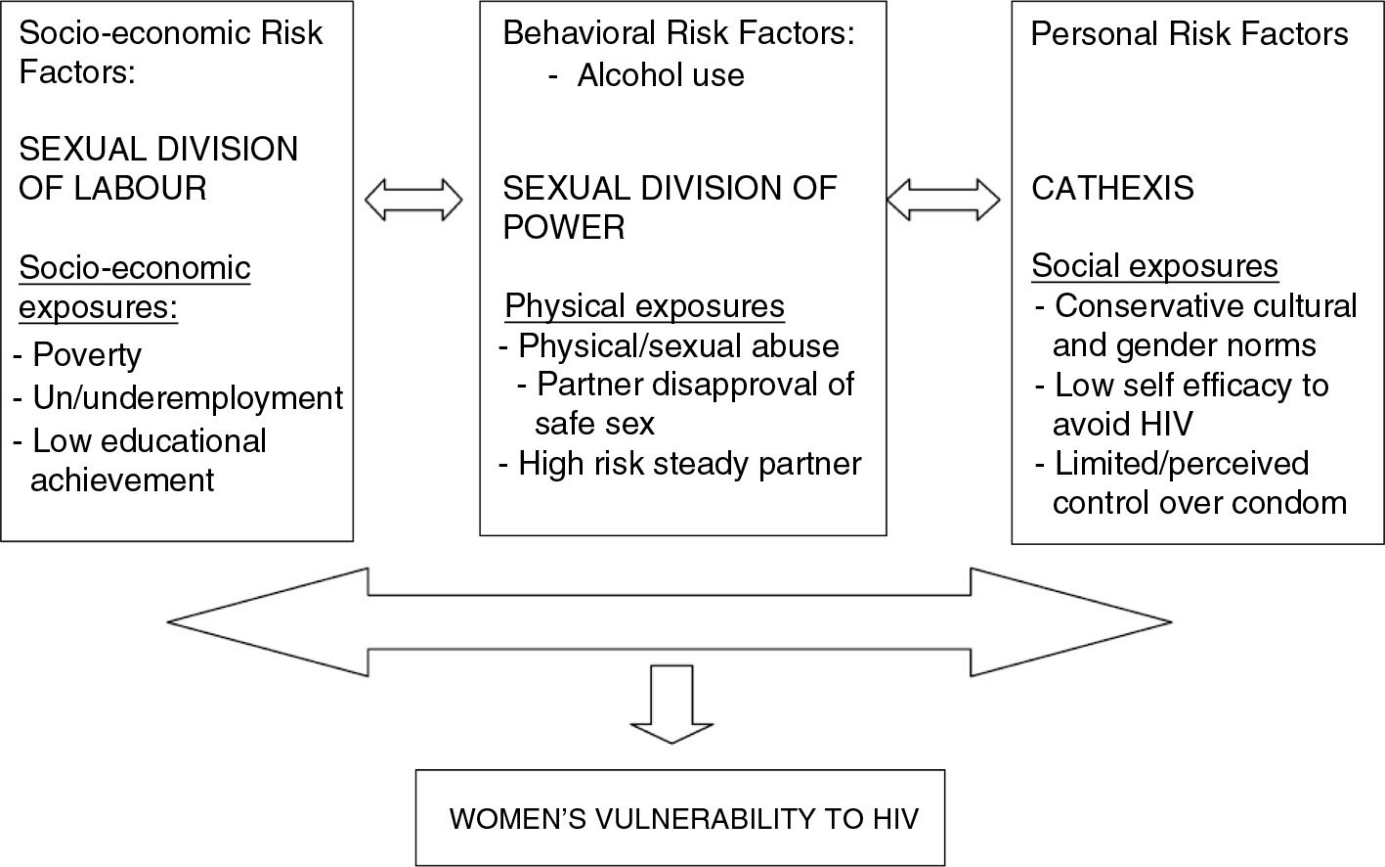
Theory of gender and power: women's exposure and risk for HIV. Adapted from Wingood and DiClemente (14).

This study applied an integrated theory of gender and power developed by Connell (13) and further extended by Wingood and DiClemente (14). Connell's theory has three basic interlinked structures, namely (a) sexual division of labor, (b) sexual division of power, and (c) the structure of cathexis (affective attachments and social norms). These structures exist both at the societal and institutional (family, work, school, and religion) levels. The three structures are absorbed and perpetuated in society through various historical and sociocultural forces that constantly isolate power and assign social norms on the basis of gender-determined roles. These social forces generate gender-based inequities in ownership and control over resources and gender-based expectations of women's role in society. Connell's theory of gender and power was further developed by Wingood and DiClemente (14) into a public health model by arguing that gender-based inequities and differences in expectations generate the exposures, or acquired risks, and the risk factors that negatively impact on the health of women. These authors argue from a public health point of view that acquired risks or exposures are associated with an increased likelihood that a disease will develop later. Furthermore, exposures can be economic (low income), physical (violence), or social (low educational achievement) in nature. This paper uses Wingood and DiClemente's public health model to facilitate an understanding of how IPV heightens sexual risk of acquiring HIV in a gender perspective (Fig. 1).

## Sexual division of labor

Socialization across cultures results in sexual division of labor whereby women engage in low-paying jobs or those that do not have monetary returns, for example, domestic chores. This pattern of socialization results in women not accessing socioeconomic opportunities such as education and employment, and they end up earning low incomes, which Wingood and DiClemente (14) refer to, in a public health context, as economic exposure to ill-health. The economic imbalance may force women to be financially dependent on men, thereby creating vulnerability to social problems or diseases related with poverty, or they may fail to leave abusive or risky relationships (14). Moreover, Wingood and DiClemente argue that the risk of the woman's health being negatively affected by sexual division of labor is increased if she belongs to an ethnic minority group or is significantly younger than her male partner.

## Sexual division of power

Connell (13) asserts that power differentials between men and women constitute a basic ingredient of gender and power theory. Besides controlling material resources and financial assets, they exercise power ‘through control, authority, and coercion within heterosexual relationships’ (15), p. 276). These power differences between the sexes are most clearly manifested through IPV, and are a central determinant of safety of sexual encounters (16). A woman may yield to non-consensual and unprotected sex after being threatened or beaten up. The current study examined pathways through which IPV exposes married women to the risk of acquiring HIV in Iringa region, Tanzania. Wingood and DiClemente (14) added that alcohol abuse enhances men's desire to manifest sexual power dominance over women. Sexual encounters happening under such circumstances increase the risk of HIV transmission as women are made to have no or low control over use of condoms.

## Cathexis

Wingood and DiClemente (14, p. 544) define the structure of cathexis as ‘societal norms and expectations regarding women's sexual behavior characterized by their sexual and emotional attachments to men’. The argument here is that gender inequities result from societal gender role prescriptions and socialization patterns. That is, women's power in intimate relations is eroded as a result of internalization of oppressive gender norms of masculinity and femininity. Married women are expected to accept and comply with their partner's practices of the ideals of masculinity such as ‘men are sexually unstoppable’ (17, 18). In essence, a ‘good’ woman is expected to internalize traditional ideals of femininity such as being submissive and tolerant. Such women are more likely to engage in risky sexual practices to please the husband (19). Failure to comply with the sexual norms may prompt the male partner to be violent. Consequently, women find themselves trapped in abusive relationships. In the current study, we examined how women are socioculturally compelled to tolerate their partners’ infidelity.

## Methods

### Design

This paper is based on a qualitative case study that involved use of focus group discussions (FGDs). The FGDs served the purpose of exploring gender dimensions surrounding involvement in sexual violence and associated risky sexual behavior prone to HIV (20–22).

### Setting

The participants in this study were drawn from the Iringa region, involving two districts, Makete and Iringa Urban, from September to October 2011. This region was selected because it had the highest prevalence of HIV (16.8%) in the country (23). In addition, Regional statistics showed that Makete, which is a rural district, had the highest HIV prevalence (16.9%) in the region followed by Iringa municipality, which is urban-based with 14.7% HIV prevalence. Besides, there were anecdotal reports that gender-based violence was on the increase in the Iringa region.

### Sampling and sample size

From each of the selected two districts, one village was selected based on the magnitude of gender-based violence as perceived by local leaders. A purposive sampling technique was used to recruit the participants. That is, focus group discussants were purposely drawn from a specific population of interest to the study – married women. In essence, being unmarried or not cohabiting were the exclusion criteria. The leaders of the select villages were approached and asked to assist in recruiting the participants. They were provided with specifications on age groups and marital status of the potential respondents. The snowball or chain sampling technique was used. This means that the first identified person(s) was/were used as a resource for identifying the next subject(s) until the required number was attained (17). A total of eight FGD sessions – consisting of 61 participants – were held. The sample size for the FGDs was determined in the field after reaching saturation (24) with respect to themes in the FGD guide. Sandelowski (25), p. 176) offers a good principle as she succinctly concludes that: ‘an adequate sample size in qualitative research is one that permits – by virtue of not being too large – the deep, case oriented analysis that is a hallmark of all qualitative inquiry, and that results in – by virtue of not being too small – a new and richly textured understanding of experience’.

### Data collection

FGD guide was used to collect the data. FGD is a qualitative research method that involved two facilitators – one being a moderator and the other a note taker (recorder) engaging a group of six to eight participants in a discussion of the research topic (Table 1). Since sexuality is a sensitive topic, as part of the introduction the moderator encouraged the participants to take part in the discussion by first expressing their understanding of the sensitivity of the topic and, second, informing them that the questions did not focus on personal experiences of sexual violence but rather on issues happening in the community at large. FGDs were chosen for use in this study because they provided an opportunity for participants to explain gender dimensions of sexual violence without personalization. That is, although the participants might have talked about their own experience, they could cover up by presenting it as something experienced by ‘others’. This is a strength of FGDs in enabling respondents to speak about sensitive information that may not easily come out with in one-to-one interviews (26, 27).

The questions that were asked included: What are the common forms of intimate partner violence experienced by married women here?; What do you consider to be sexual violence in marriage?; What are the factors contributing to sexual violence in marriage? (probes role of disparities in ownership of resources between spouses and power disparities in marital sexual relationships); What societal expectations regarding women's sexual behavior increases their risk of contracting HIV in marriage?

The data were collected in Kiswahili, transcribed verbatim, and then translated into English.

**Table 1:** Characteristics of the FGD participants

	Characteristic	Number (*N*=61)
1	Age groups	
	18–25	14
	26–35	47
2	Occupation	
	Peasantry	44
	Peasantry and small-scale business	17
3	Education	
	Never been to school	12
	Primary level of education	49

### Data analysis

A thematic analysis approach was used in analyzing the study findings. The English translated data were analyzed through the examination and categorization of respondents’ opinions. The analysis was carried out in three stages (28, 29): first, the line-by-line coding of field notes and transcripts; second, the in-depth examination and interpretation of the resultant codes and their categorization into descriptive and analytical themes; and third, the development of an overarching theme. The coding involved the development of concepts – that is, the data were parsed into discrete elements in order to expose underlying thoughts and meanings. The process generated 38 codes, which were further interpreted and categorized into eight descriptive codes. These latter codes were further distilled into four abstract analytical themes around which results are presented. Table 2 illustrates how the analytical themes were obtained.

**Table 2:** An illustration of line-by-line coding and development of analytical themes

Text	Codes	Descriptive themes	Analytical themes
‘The husband is everything in the house. He brings food and pays for basic needs in the family. Thus, when it comes to sex, he demands rather than requesting. He sees that all financial expenditure that he does for the welfare of his wife and the family should be acknowledged by being granted access to sex as he wishes. The wife's decision not to comply may trigger her husband's anger and she agrees to have non-consensual sex in order to avoid impending physical violence’.	Male dominance Bread earner Financial support to the woman Financial support to the family Sexual demands Demand for sexual appreciation Non-compliance trigger anger Non-consensual sex Avoidance of physical violence Sex as per husband's wish	Husband is economically dominant The woman depends on her husband for food and other basic needs The woman is compelled to render sexual service to her husband in return for financial/social support from him.	Economic powerlessness exposes women to violence
The man does not agree to be denied his marital right to sex, he forces even if you are tired or sick, he asks: ‘what did I marry you for? I paid the pride price to your family’. He threatens because he owns you	Man's marital right to sex No regard for the woman's concerns Sex: a reason for marriage Culture of paying bride price Bride price grants ‘ownership’ Threats	The woman is perceived as a ‘purchased’ sexual object The woman is forced into submission	Husband assumes ownership of his wife
In this community, men are fond of taking alcohol. A man who goes to alcohol clubs may easily be tempted to engage in extramarital sexual relationships. And when he does so and his wife happens to know she may resist having sex with him. That is when the husband decides to have sex forcefully	Alcohol highly valued by men Men indulge in alcohol drinking Alcohol clubs are meeting places Drunkard men likely to initiate and engage in extramarital sexual affairs Women's resistance to Drunkard men's sexual advances Forced sex	Alcohol aids in masculine identity construction. The state of drunkenness pushes men to initiate and engage in extramarital sexual affairs	Synergy between masculinity and alcohol consumption
Even if you know that your husband has an extramarital sexual partner, it is very difficult for the wife to refuse not to have sex with him. Eeh, it is his right. It is the matrimonial vows that keep us trapped. As a woman you are obliged to obey your husband. Therefore, you have to agree to have sex even though grudgingly – otherwise marriage may breakdown	High-risk husband Inability to refuse to have sex The role of matrimonial vows Obedience expected Sexual compliance out of necessity Non-consensual sex Threat of marriage breakdown	The woman is unable to refuse to have sex with a high-risk husband A good woman is expected to be tolerant and obedient to her husband under all conditions	Compelled tolerance of high-risk sex in marriage

The two authors coded the transcripts and generated descriptive and analytical themes independently. Then, the corresponding author convened several meetings during which the codes and themes generated by each author were compared. There was little deviation from each other's findings, and the two authors jointly agreed on analytical themes presented in this paper.

### Trustworthiness

The trustworthiness of the results can be secured through several ways such as prolonged engagement, members’ checks, and peers debriefing (24). In this study, the FGD guide was piloted to improve the questions and the moderating skills. The data collection period was planned such that the researchers had enough time for reflection between field visits and were therefore able to conduct preliminary analyses that guided their subsequent data collection. Moreover, a member check technique was applied during group discussion whereby the moderator restated or summarized the information from the discussant(s) to ensure what was heard was in fact correct.

Besides, this paper benefited from the investigator triangulation whereby both authors took part in data analysis, first independently and then jointly. The findings presented in this paper arose from the consensus between the two authors.

The authors are therefore confident that the findings are valid and grounded in the data.

### Ethical considerations

The ethical clearance for this study was obtained from Muhimbili University of Health and Allied Sciences (MUHAS). The prospective FGD participants were requested to participate in the study. Before consenting, the moderator told the participants that their participation was purely voluntary. Furthermore, they were informed that no names were required and data would be treated with high level of confidentiality. Moreover, participants were requested to also maintain confidentiality by not revealing personal experiences that might feature in the discussion to other people who did not participate in it. Finally, each potential participant was informed of her right to refuse to participate. They provided an oral consent.

## Results

Presentation of results is guided by three structures of the above-described theory of gender and power. Under each structure, results are presented around the analytical theme(s) that emerged from analysis.

### Sexual division of labor: economic powerlessness exposes women to violence

The structure of sexual division of labor argues that the economic imbalance forces women to be financially dependent on men, thereby making them vulnerable to social problems, and they may fail to leave abusive or risky relationships. Under this structure, analyses of data resulted in one analytical theme: *economic powerlessness exposes women to violence*. Our study respondents argued that men control most resources with concomitant responsibility to enable their wives and children to meet basic needs such as food, shelter, and clothing. Further to that, the economic power that men have over women makes them [men] think that women have an obligation to provide sexual ‘service’ to them. Consequently, if it happens that a wife declines her husband's request for sexual ‘service’, the latter may feel justified to force his partner into submission. One of the FGD participants thus said:

The husband is everything in the household. He brings food and pays for basic needs in the family. Thus, when it comes to sex, he demands rather than requesting. He sees that, all financial expenditure that he does for the welfare of his wife and the family should be acknowledged by being granted access to sex as he wishes. The wife's decision not to comply may trigger her husband's anger and she agrees to have non-consensual sex in order to avoid impending physical violence. (Woman, 34 years, Makete)

### Sexual division of power: men coerce women into having sex

The structure of sexual division of power focuses on power differences between spouses. It is argued that men exert power through coercion within heterosexual marriage. Under this structure, analyses of our data resulted in two themes: husband assumes ownership of his wife, and synergy between masculinity and alcohol consumption.

#### Husband assuming ownership of his wife

The findings of our study show that the tendency of wanting to demonstrate a more powerful position in marriage emanates partly from the way marriage is perceived in a patriarchal society. The notion of marriage whereby a man pays the pride price and the female partner moves from her home or her parents’ home to the husband's home implies power differences/disparities between the spouses. That is, a man tends to believe that he has the decision-making power over his partner on many aspects including sexual acts. It is the husband who takes a lead on deciding when and how to have sex. Indeed, in-depth analysis has revealed that men consider their wives to be sexual objects. This perspective was very well illustrated by one focus group discussant:

A man does not agree to be denied his marital right to sex, he forces even if you are tired or sick, he asks: ‘what did I marry you for? I paid the pride price to your family’. He threatens because he owns you. (Woman, aged 28 years, Makete)

Similarly, the study respondents identified men's perception of the role of women in sexual relationships as an important factor influencing sexual violence. It was argued that many times men are preoccupied with the desire to attain sexual gratification and expect women to comply without regard to related consequences. As a result, the study respondents pointed out that a woman is forced by her partner into having sex even on ovulation day – when the woman is highly likely to become pregnant. One of the participants said, ‘the man just cares about getting sexual pleasure. So he would persistently persuade you to have sex with him even when you are very likely to conceive. He does not care’ (Woman, aged 42 years, Iringa).

#### Synergy between masculinity and alcohol consumption

Views from the study participants suggested that there is a synergistic relationship between masculinity notions of dominance and alcohol consumption. It was argued that men tend to affirm their masculinity by drinking alcohol which reinforces the notions of a ‘real man’ – fearless, risk taker, and sexually unstoppable. Alcohol emerged strongly as a factor that fuels non-consensual sex. On the other hand, the influence of alcohol causes the man to underestimate the risk arising from his masculine decisions and actions. It was thus argued that alcohol consumption is the genesis of other evils that follow from being drunk: increased lust for extramarital sexual partners; inability to effectively use condoms; forcing the marital partner to engage in unprotected sex. One of the focus group discussants thus remarked:

In this community, men are found of taking alcohol. A man who goes to alcohol Clubs may easily be tempted to engage in extramarital sexual relationships. And when he Does so and his wife happens to know she may resist having sex with him. That is when the husband decides to have sex forcefully. (Woman, aged 35 years, Iringa)

### Cathexis: compelled tolerance of high-risk sex in marriage

The structure of cathexis refers to societal norms and expectations regarding women's sexual behavior characterized by their sexual and emotional attachments to men. Under this structure, analysis of our data resulted in one theme: compelled tolerance of high-risk sex in marriage.

Our study respondents argued that a married woman is expected to be obedient under all conditions. Thus, it was argued that although there are women who genuinely suspect that their husbands are unfaithful, they feel obliged to continue fulfilling their husbands’ sexual demands – albeit with a broken heart – for the sake of maintaining marriage.

Even if you know that your husband has an extramarital sexual partner, it is very difficult for the wife to refuse not to have sex with him. Eeh, it is his right. It is the matrimonial vows that keep us trapped. As a woman you are obliged to obey your husband. Therefore, you have to agree to have sex even though grudgingly – otherwise marriage may breakdown. (Woman, aged 25 years, Iringa)

It was further argued that because of the expectation that the woman should be obedient and submissive, she finds it hard to ask that a condom be used for protection against potential HIV transmission from her unfaithful husband. Under such circumstances, demanding that condoms be used during sex amounts to confirming allegations of infidelity against her husband – something that may trigger physical and/or emotional violence. One focus group discussant reported that a man may object to his wife's request by saying: ‘I cannot use a condom because you are my wife, don't you trust me?’ (Woman, aged 25 years, Iringa).

## Discussion

This section discusses main study findings presented under the three structures of gender and power theory. *On sexual division of labor*, our study found that economic powerlessness exposes women to sexual violence. This suggests that the economic power that men have over women gives them the status of a bread warner and an expectation that his wife fully complies with his sexual advances in return. When this expectation is not met, a man may become violent physically and/or emotionally and force his partner into submission to having non-consensual sex. Similar findings have been reported by ICASO (30).

*On sexual division of power*, our study found that perception of the man as a more powerful partner in marriage is enhanced by the biased marriage arrangement and alcohol consumption. On marriage arrangement, the culture of paying pride price was blamed for making the man assume ‘ownership’ of his wife. There is logic in this argument. Paying the pride price amounts to turning the woman into an object that is bought from the market and owned by the buyer. In effect, the ‘transaction’ diminishes the woman's freedom and power of resisting abuse by the husband.

Our study's findings are in agreement with those reported in Uganda and elsewhere in Africa (31–34). These authors argue that bride price influences men's aggression tendency and make women lose their dignity – thereby increasing their vulnerability to sexual abuse and HIV infection.

Besides, views of our study participants suggested that there is a synergistic relationship between masculinity notions of dominance and alcohol consumption. This implies that while the masculinity notions of a *real man – fear less, risk taker, and sexually unstoppable* – are potentially likely to influence the man toward having non-consensual sex with his partner, drinking alcohol ‘energizes’ the behavior by providing a sense of legitimacy through its inhibitory effect on the conscience. Previous studies (35, 36) have documented that there is a very close interrelationship between masculinity and consumption of alcohol. The linkage between alcoholic drinking and sexual abuse as well as sexual risk behavior in marriage has also been demonstrated in previous works (37–43). Despite this accumulating evidence, clear national health strategies related to the role of alcohol in HIV/AIDS transmission are virtually lacking (44). The synergy between masculinity and alcohol drinking needs to be considered when developing interventions against sexual abuse and sexual risk behaviors among married women.

*On cathexis*, this study has revealed that because of societal norms and expectations regarding women's sexual behavior characterized by their sexual and emotional attachments to men, women find it hard to leave sexually abusive marriages. That is, because of societal expectations of obedience and tolerance many married women do suffer in silence. They find themselves like they are trapped in marriages that increase their risk of acquiring HIV. This might explain why there are many married women who have been infected with HIV (45). Despite knowing that their husbands have high-risk sexual behaviors, including having multiple sexual partners, they choose to compromise and tolerate because of gender-related psychological subordination. Similar findings have been reported in Nepal (46).

This study has demonstrated that Wingood and DiClemente's framework is a useful tool for a structured and systematic understanding of how gender imbalances influence sexual violence and the risk of acquiring HIV among married women in Africa. Although Wingood and DiClemente's definitions of exposures and risk factors were conceived in the American context, they are adaptable to the African situation. For instance, on the sexual division of labor, Wingood and DiClemente's framework identifies one of the socioeconomic risk factors as an ethnic minority woman such as Latino or Black American. In Africa, and particularly in Tanzania, the problem is generally common across all societies. That is, married women in almost all African societies are vulnerable to sexual violence as a result of sexual division of labor for two reasons: first, economic dependence on male partners/feminization of poverty cuts across cultures, and second, coercing marital partners into having sex even when they do not want to is a common feature among African men as part of their social construction of masculinity and implicit ‘demand for compensation’ in return for the economic support they render to the family (47, 48).

### Limitations of the study

This study is not without limitations. First, since it used a purposive sampling technique, it had inadequate representativeness and thus findings cannot be generalized. However, being a qualitative study, its goal was not to generalize but rather to provide rich information on gender context of sexual violence and associated HIV sexual risk marriage. Second, the findings of our study should be interpreted in the light of the strengths and weaknesses inherent to the use of group-based data collection methods. Whereas FGDs are useful for exploring common concerns from discussants, there are limitations on what individual group members can openly express in the presence of others particularly so on sensitive topics like sexuality (49). However, the researchers addressed this drawback by ensuring that the FGDs were conducted in an ethical manner. For instance, the moderator assured the FGD participants of confidentiality by telling them that no names will be used in the report or publications resulting from the study. The participants were further asked to protect the privacy of others in the group by not discussing personal information shared during the FGD with other community members who did not participate.

## Conclusion and recommendations

This study suggests that married women experience a sexual risk of acquiring HIV that results from non-consensual sex. That non-consensual sex is a function of gender imbalances – women's economic dependence on their husbands; women perceived as sexual objects because of the bride price; masculinity notions of dominance fueled by alcohol consumption; and socioculturally rooted norms and expectations regarding women's sexual behavior that make them hesitant to leave sexually abusive marriages. The HIV risk is especially heightened because masculinity sexual norms encourage men (husbands) to engage in unprotected intra- and extramarital sex.

Based on the findings of this study, it is recommended that: The HIV and AIDS control program in Tanzania may use the theory of gender and power as a framework for conducting gender analysis and eventually develop both practical and strategic interventions toward reducing transmission of HIV among married women and their partners. Tentatively, the following actions are suggested. Tanzania Commission for AIDS (TACAIDS) and human rights organizations should address the question of sexual violence in marriage; there is need to initiate community-based educational programs for sensitizing people against masculine attitudes that negatively impact on sexual health; there is a need to have a couples-counseling program for healthy sexual relationships in marriage; since many women suffer in silence, it is important that health providers inquire about sexual violence in marriage during routine visits by women – so that protective measures can be instituted.
